# Correction to ‘Identification of differentially expressed genes in a resistant versus a susceptible blueberry cultivar after infection by *Colletotrichum acutatum*’

**DOI:** 10.1111/mpp.13495

**Published:** 2024-07-09

Miles, T.D., Day, B., Schilder., A.C. Identification of differentially expressed genes in a resistant versus a susceptible blueberry cultivar after infection by *Colletotrichum acutatum*. *Mol. Plant Pathol*., 2011; 12:463‐477.

Figure 5 has been updated because there was an unintentional cropping error in two of the gel images within the previous Figure. This has been corrected in the below image.

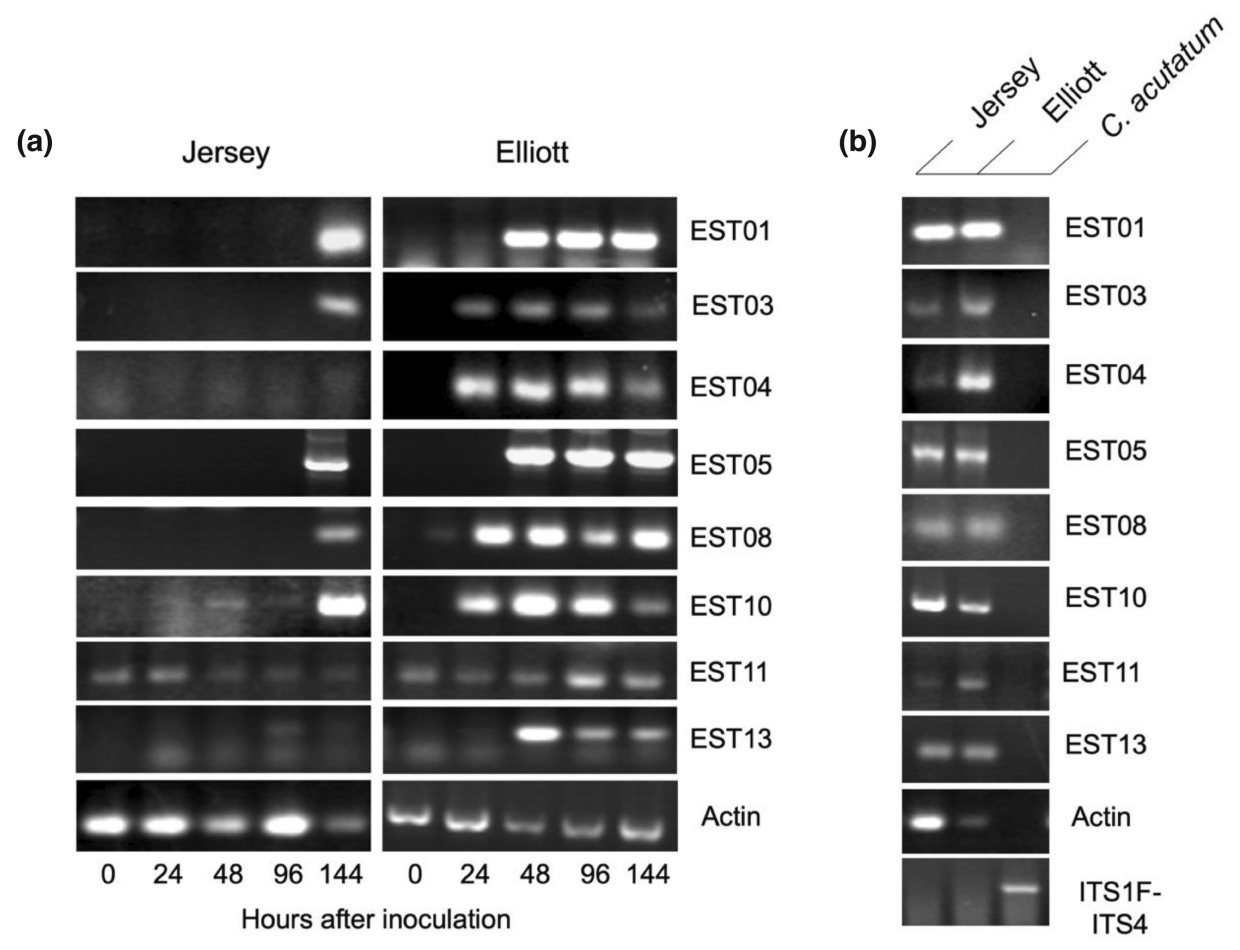

We apologize for this error.

